# Supplementary material for: MVP predicts the pathogenicity of missense variants by deep learning
Source: Nat Commun. 2021 Jan 21;12:510. doi: 10.1038/s41467-020-20847-0 (PMC7820281; doi:10.1038/s41467-020-20847-0)
Supplement: Supplementary file 1 — Supplementary Information [file 41467_2020_20847_MOESM1_ESM.pdf]

Supplementary Information

MVP predicts pathogenicity of missense variants by deep learning

Qi H, Zhang H, Zhao Y, Chen C, *et. al.*

Table of Contents

Supplementary Figures ***2***

Supplementary Figure 1 .... **2**

Supplementary Figure 2 .... **3**

Supplementary Figure 3 .... **4**

Supplementary Figure 4 .... **5**

Supplementary Figure 5 .... **6**

Supplementary Figure 6 .... **7**

Supplementary Figure 7 .... **8**

Supplementary Figure 8 .... **9**

Supplementary Figure 9 .... **10**

Supplementary Figure 10 .... **10**

Supplementary Figure 11 .... **11**

Supplementary Figure 12 .... **12**

Supplementary Figure 13 .... **13**

Supplementary Figure 14 .... **14**

Supplementary Figure 15 .... **14**

Supplementary Figure 16 .... **15**

Supplementary Figure 17 .... **16**

Supplementary Figure 18 .... **17**

Supplementary Figure 19 .... **17**

Supplementary Table 1 .... **18**

Supplementary Table 2 .... **19**

Supplementary Note 1 .... ***20***

Supplementary Figures

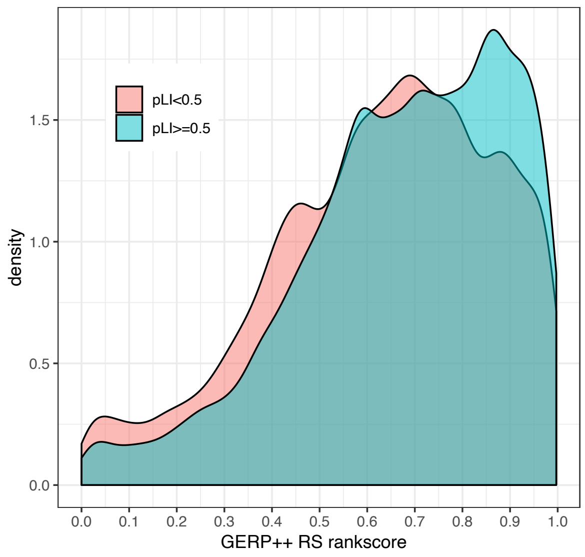

Supplementary Figure 1. Distribution of GERP++ RS score in training set. Pathogenic variants in constrained genes (pLI≥0.5) are likely to be more conserved than the ones in non-constrained genes (pLI<0.5).

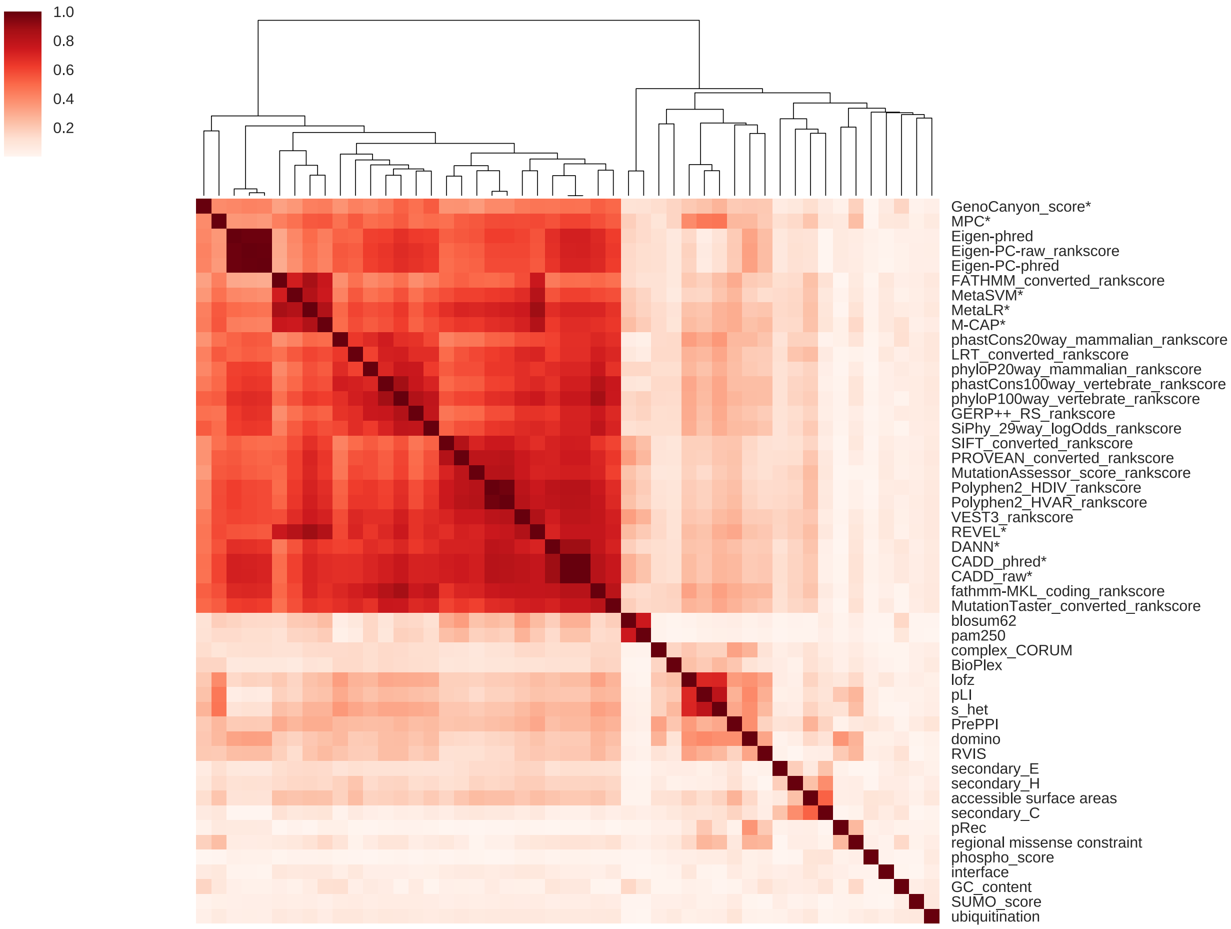

Supplementary Figure 2. Correlation and hierarchical clustering of features and additional published methods. We calculated pairwise Spearman correlation of all features and additional published methods across data points used in the training**.** Color key indicates absolute value of Spearman correlation coefficient among features and predictors. Columns are ordered by hierarchical clustering. Published methods marked with * are not used in training.

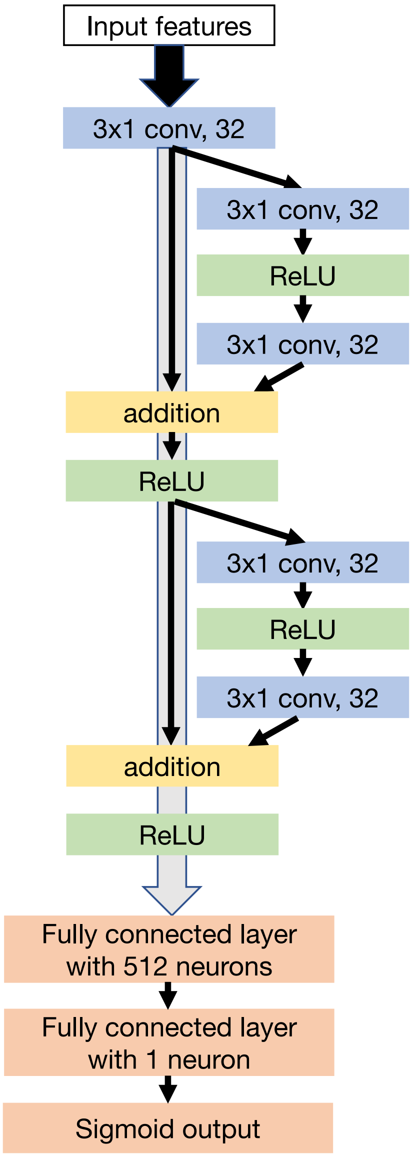

Supplementary Figure 3. The ResNet neural network architecture of MVP. Building blocks are arranged as shown in the figure. Parameters and dimensions of input and output are indicated in the boxes. Blue boxes are convolutional filters, green boxes are ReLU activation, yellow boxes are addition of outputs from 2 layers, orange boxes are fully connected layers.

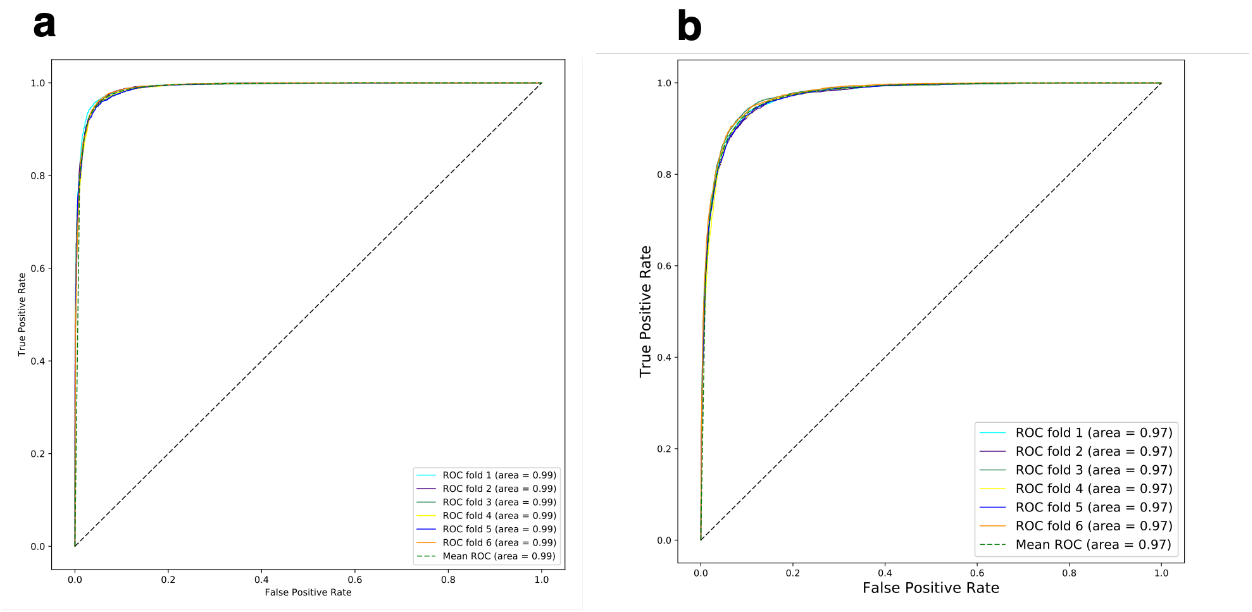

Supplementary Figure 4. Receiver operating characteristic (ROC) curves of MVP with 6-fold cross validation in the training dataset. **(a)** Performance evaluation in constrained genes (ExAC pLI ≥ 0.5). **(b)** Performance evaluation in non-constrained genes (ExAC pLI < 0.5). The performance of MVP in each fold is evaluated by the ROC curve and Area Under Curve (AUC) score indicated in parenthesis. Higher AUC score indicates better performance.

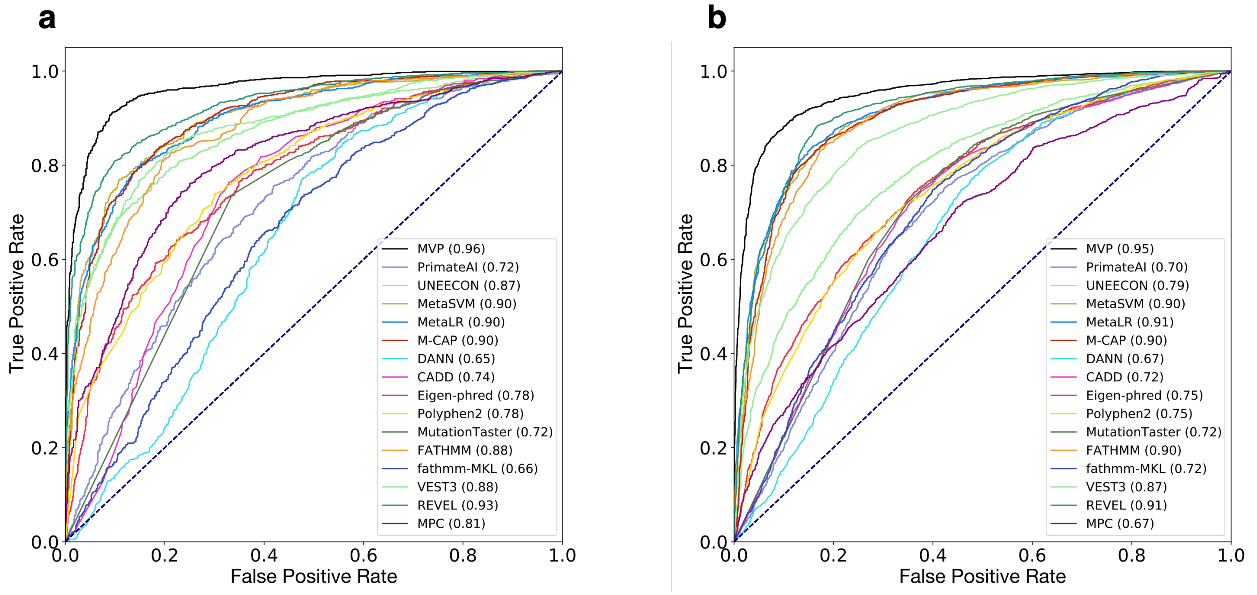

Supplementary Figure 5. Comparing MVP with previous methods by ROC curves using VariBench testing data. (a) Performance evaluation in constrained genes. **(b)** Performance evaluation in non-constrained genes. The performance of each method is evaluated by the ROC curve and AUC score indicated in parenthesis. Higher AUC score indicates better performance.

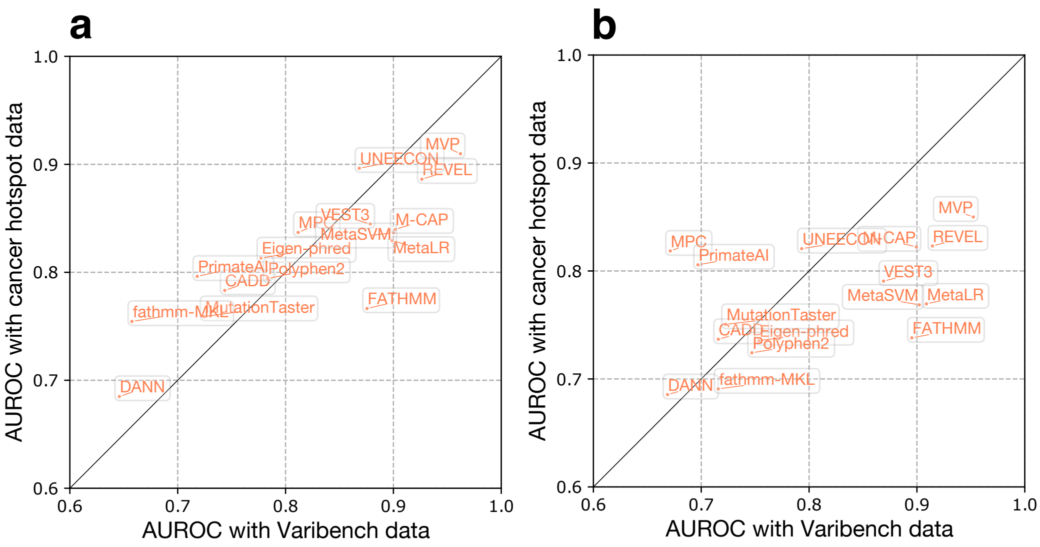

Supplementary Figure 6. Comparison of AUC using VariBench data versus cancer mutation hotspots data for MVP and previous methods. X-axis indicates the AUC with VariBench data; y-axis indicates the AUC with cancer hotspots data. **(a)** comparison in constrained genes (ExAC pLI ≥ 0.5). **(b)** comparison in non-constrained genes (ExAC pLI < 0.5).

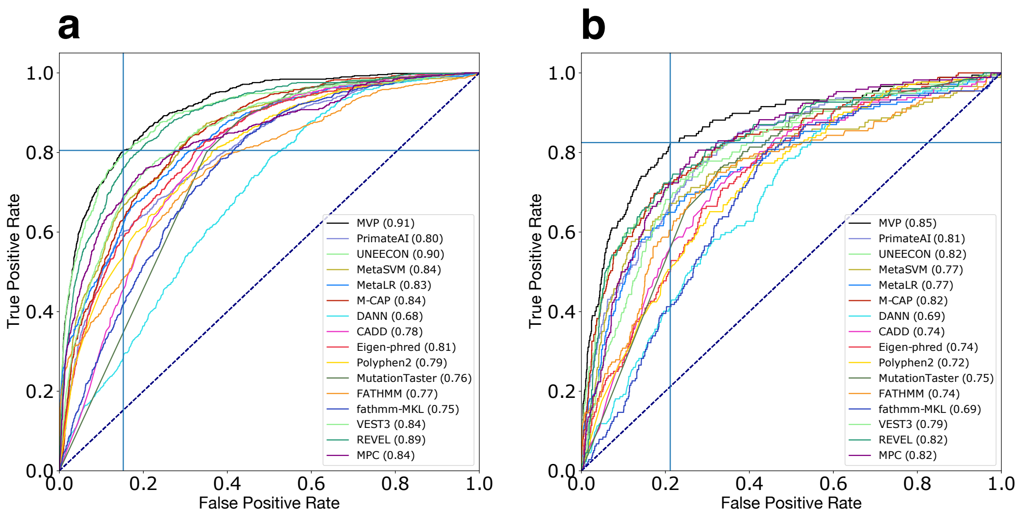

Supplementary Figure 7. Optimal threshold of MVP score based on ROC curve using cancer somatic mutation hotspots data. Horizontal line and vertical line indicated the optimal threshold in which the ROC curve has the maximum distance to the diagonal line; **(a)** Constrained genes (ExAC pLI ≥ 0.5): MVP score 0.7 is best threshold; **(b)** Non-constrained genes (ExAC pLI < 0.5): MVP score 0.75 is best threshold.

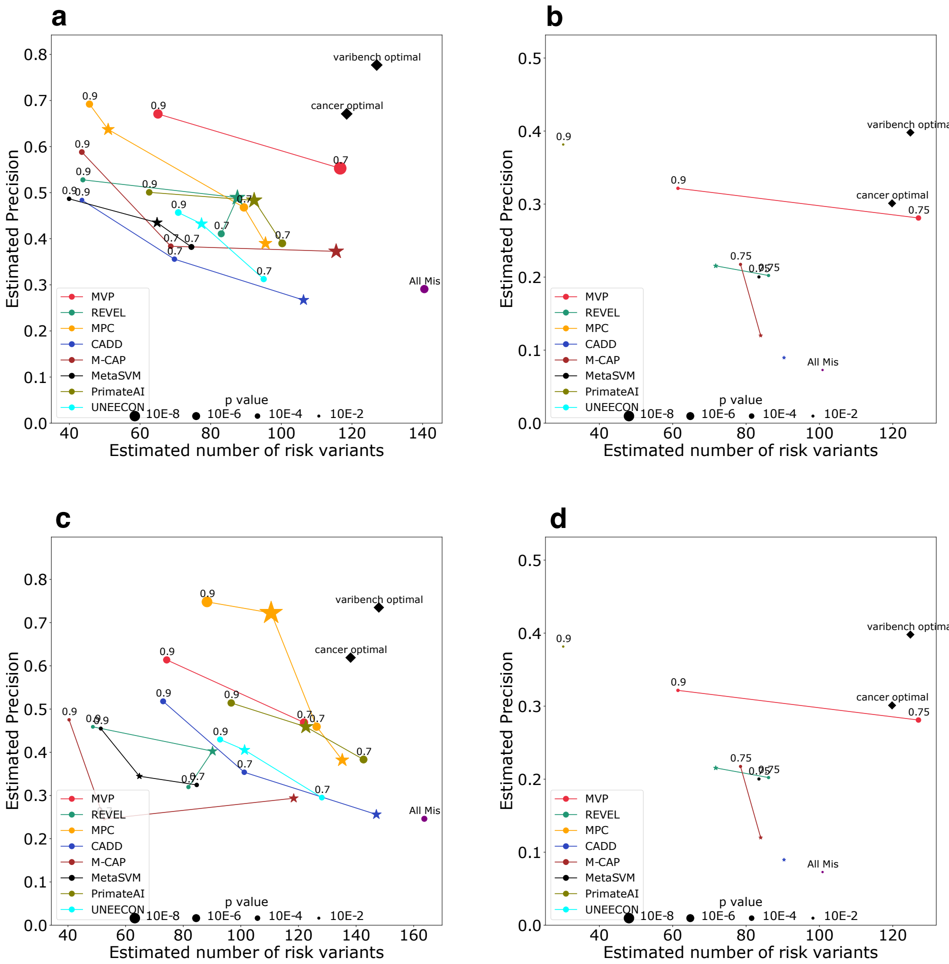

Supplementary Figure 8. Comparison of MVP and previous methods using *de novo* missense mutations from CHD and ASD studies by precision-recall-like curves. Numbers on each point indicate rank percentile thresholds; star points indicate thresholds recommended by publications. The position of "All Mis" points are estimated from all missense variants in the gene set without using any pathogenicity prediction method, ***black diamonds*** indicate estimated precision and number of variants from cancer hotspot ROC curve and VariBench ROC curve. The size of each point is proportional to –log(p-value). P-value is calculated by two-sided Binomial test, and only points with p-value < 0.05 are shown. **(a, b)** Performance in CHD *de novo* data in constrained genes and non-constrained genes, respectively. **(c, d)** Performance in ASD *de novo* data in constrained genes and non-constrained genes, respectively.

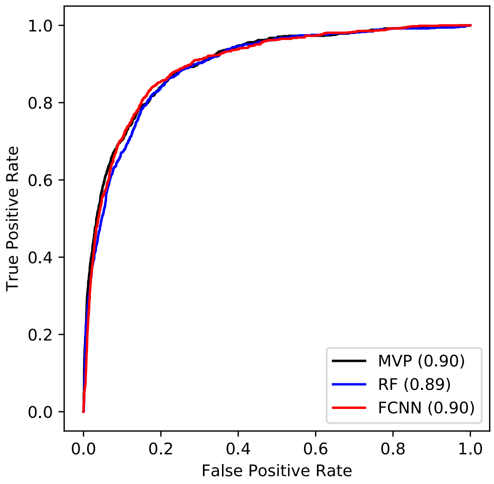

Supplementary Figure 9. ROC curves for Random Forest, Fully-connected Neural Network and MVP scores of cancer somatic mutation data sets. There are 875 variants in cancer hotspots and 8771 variants randomly selected from DiscovEHR database.

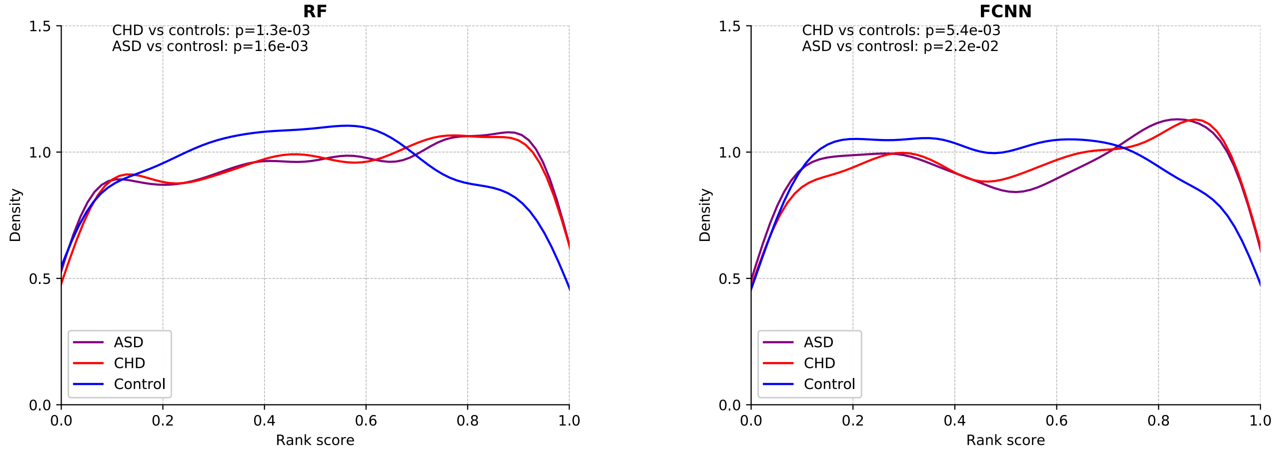

Supplementary Figure 10. Distribution of predicted scores of *de novo* missense variants by Random Forest and Fully-connected Neural Network. For each method, we normalized all predictions by rank percentile, and used two-sided Mann–Whitney U test to assess the statistical significance of the difference between cases and controls.

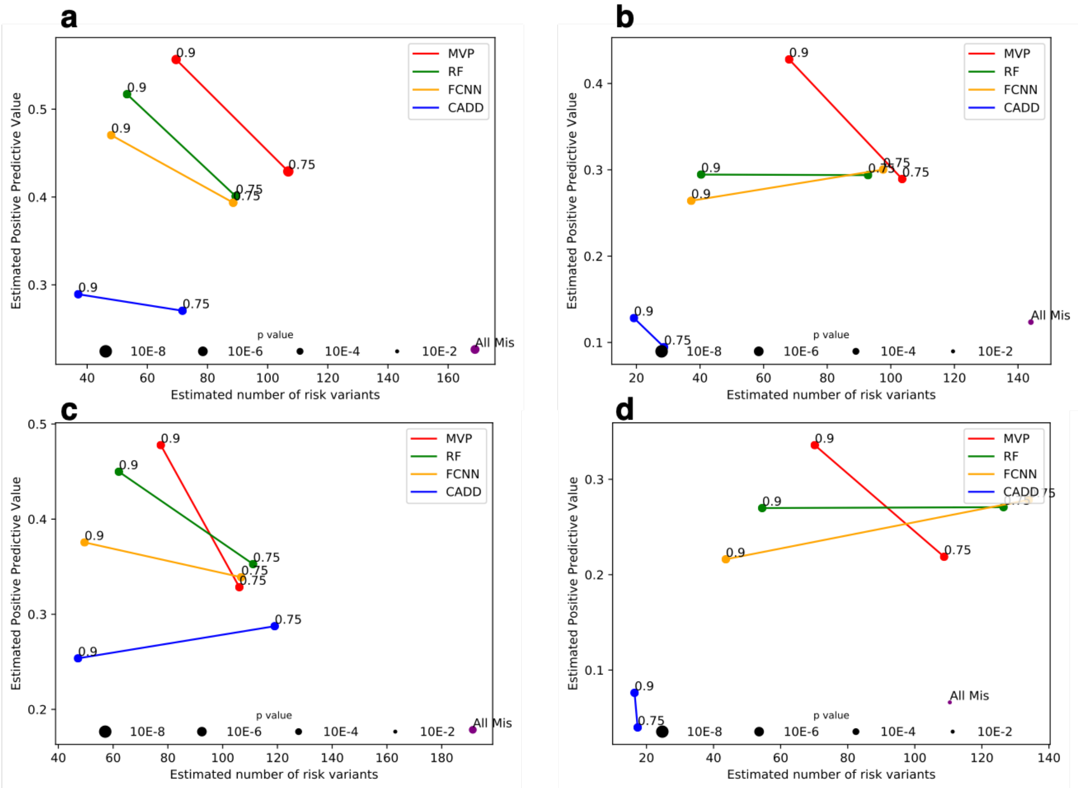

Supplementary Figure 11. Comparison of MVP, Random Forest, and Fully-connected Neural Network using *de novo* missense mutations from CHD and ASD studies by precision-recall-like curves. Numbers on each point indicate rank percentile thresholds; star points indicate thresholds recommended by publications. The position of "All Mis" points are estimated from all missense variants in the gene set without using any pathogenicity prediction method. The size of each point is proportional to –log(p-value). P-value is calculated by two-sided Binomial test, and only points with p-value < 0.05 are shown. **(a, b)** Performance in CHD *de novo* data in constrained genes and non-constrained genes, respectively. **(c, d)** Performance in ASD *de novo* data in constrained genes and non-constrained genes, respectively.

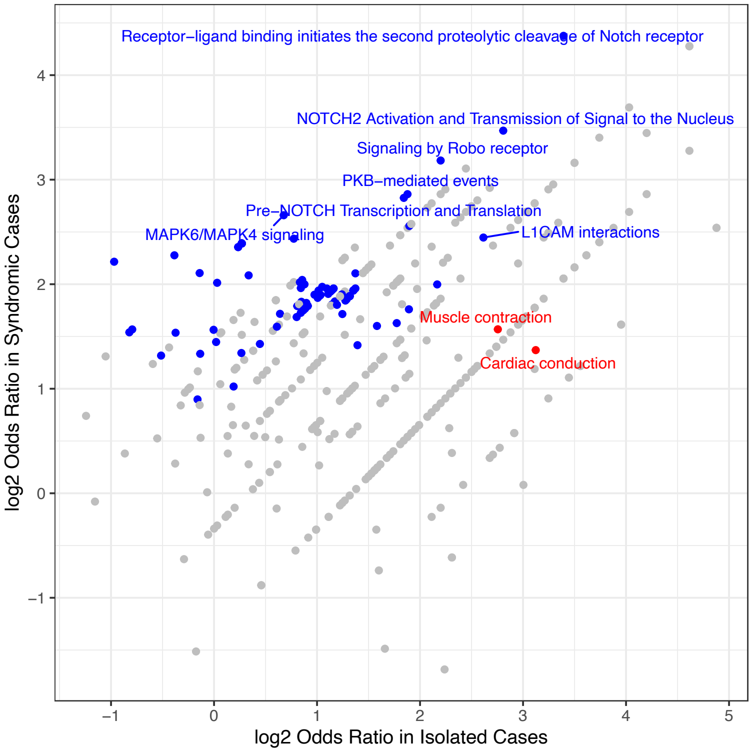

Supplementary Figure 12. Pathway enrichment of genes with MVP-predicted pathogenic variants in CHD cases. Results are given by Enrichr using Reactome database. 136 genes in isolated CHD cases and 344 genes in syndromic cases with *de novo* missense variants of MVP rank score ≥ 0.75 are used respectively. Red: pathway with FDR < 0.01 in isolated cases; Blue: pathway with FDR < 0.01 in syndromic cases.

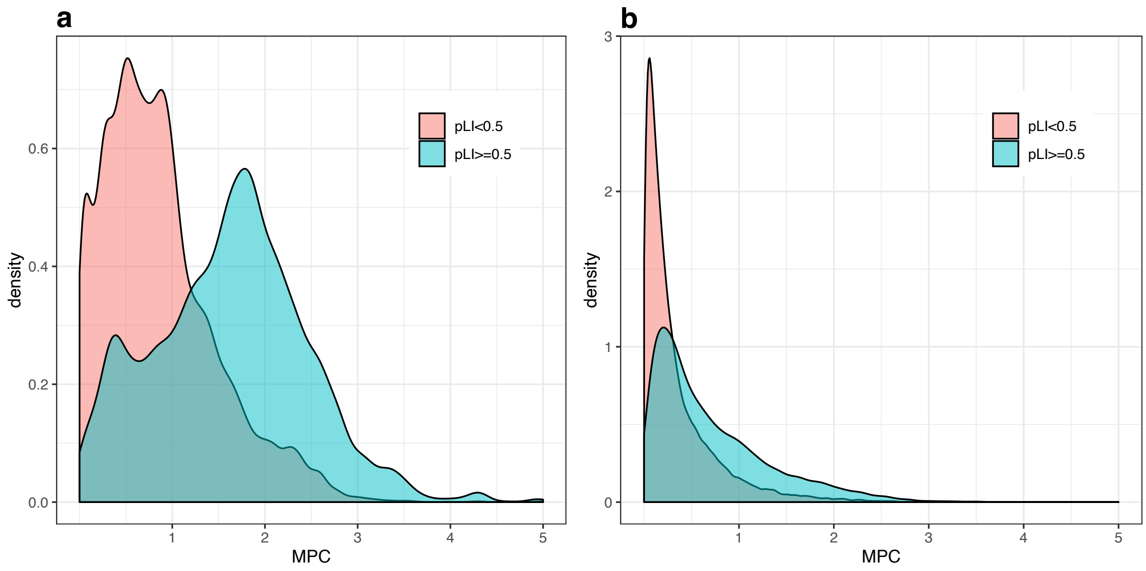

Supplementary Figure 13. Distribution of MPC scores on training data set. Variants in constrained genes (pLI>=0.5) have higher MPC scores than those in non-constrained genes (pLI<0.5), either for **(a)** pathogenic variants or **(b)** benign variants.

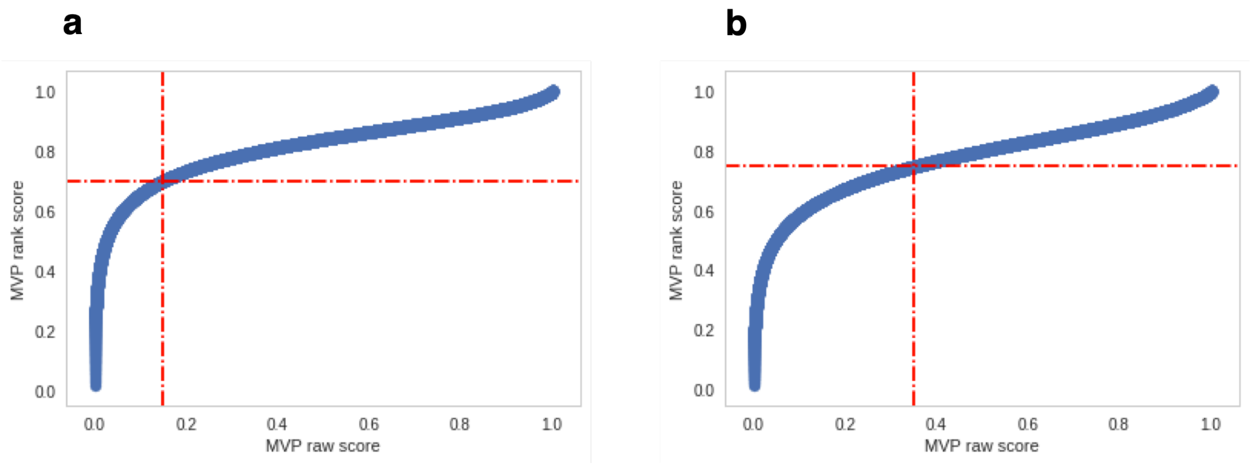

Supplementary Figure 14. Transformation between MVP rank scores and MVP raw scores. Dashed lines indicate optimal threshold. **(a)** Constrained genes (ExAC pLI ≥ 0.5); **(b)** non-constrained genes (ExAC pLI < 0.5).

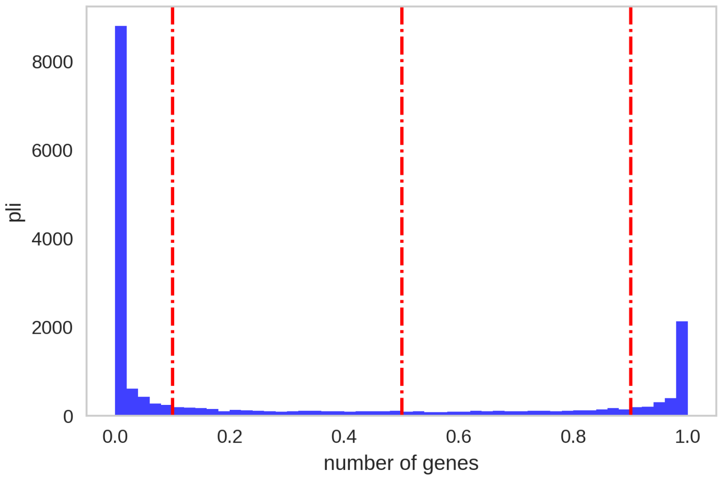

Supplementary Figure 15. **Distribution of pLI score among all genes.** Dashed lines represent thresholds of pLI = 0.1, 0.5, 0.9. Most genes have a pLI score > 0.9 or < 0.1.

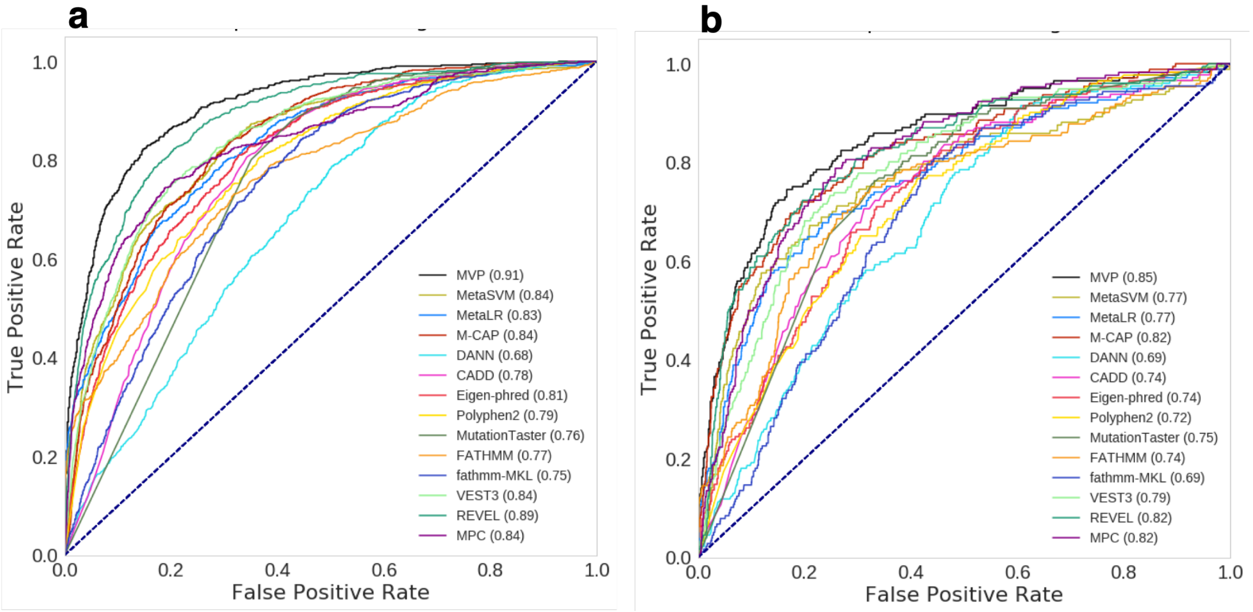

Supplementary Figure 16. Performance of MVP score on ROC curve using cancer somatic mutation hotspots data by different pLI cutoffs. (a) Performance in constrained genes (ExAC pLI ≥ 0.5) of MVP model trained by variants in genes with pLI ≥ 0.9; **(b)** Performance in non-constrained genes (ExAC pLI < 0.5) of MVP model trained by variants in genes with pLI ≤ 0.1.

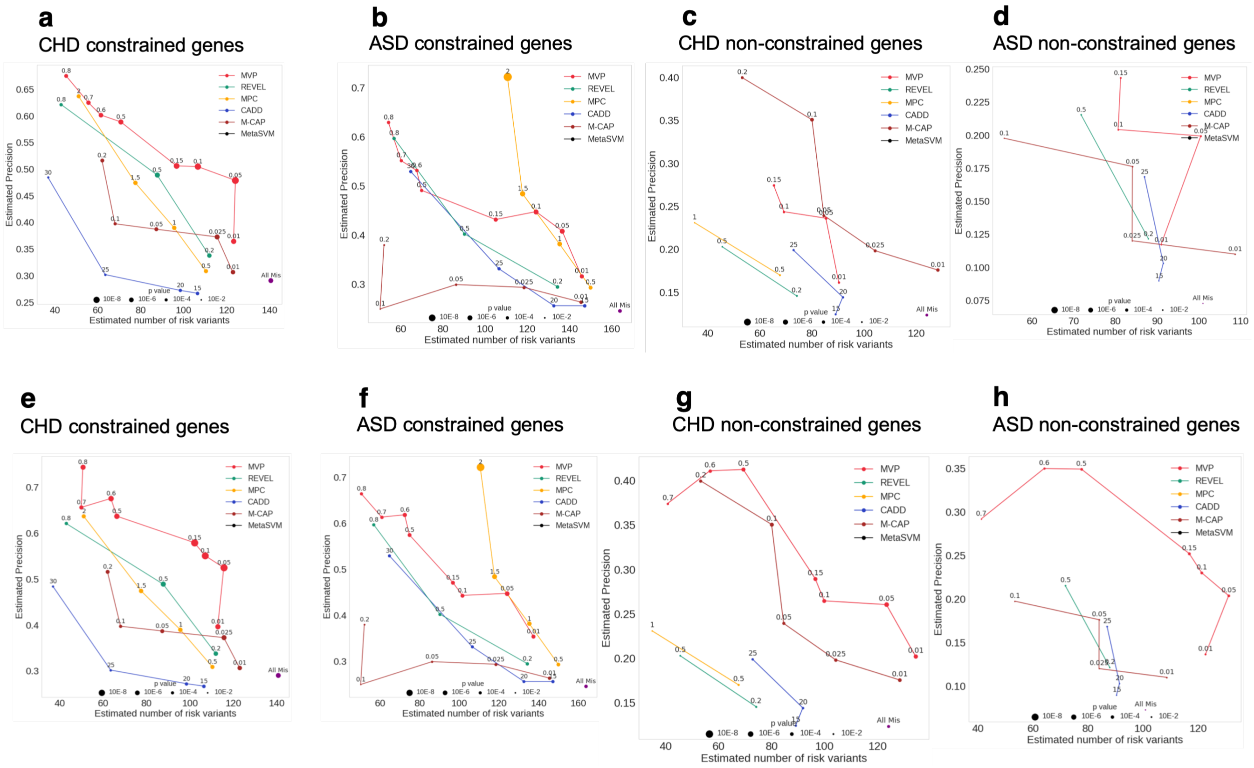

Supplementary Figure 17. Comparison of MVP by different gene sets and previous methods using *de novo* missense mutations from CHD and ASD studies by precision-recall-like curves. Numbers on each point indicate raw scores. The position of "All Mis" points are estimated from all missense variants in the gene set without using any pathogenicity prediction method. The size of each point is proportional to –log(p-value). P-value is calculated by two-sided Binomial test, and only points with p-value < 0.05 are shown. **(a, b, c, d)** MVP trained using all genes. Performance in CHD/ASD *de novo* data in constrained genes and non-constrained genes, respectively. **(e, f, g, h)** MVP trained using constrained gene and non-constrained genes separately. Performance in CHD/ASD *de novo* data in constrained genes and non-constrained genes, respectively.

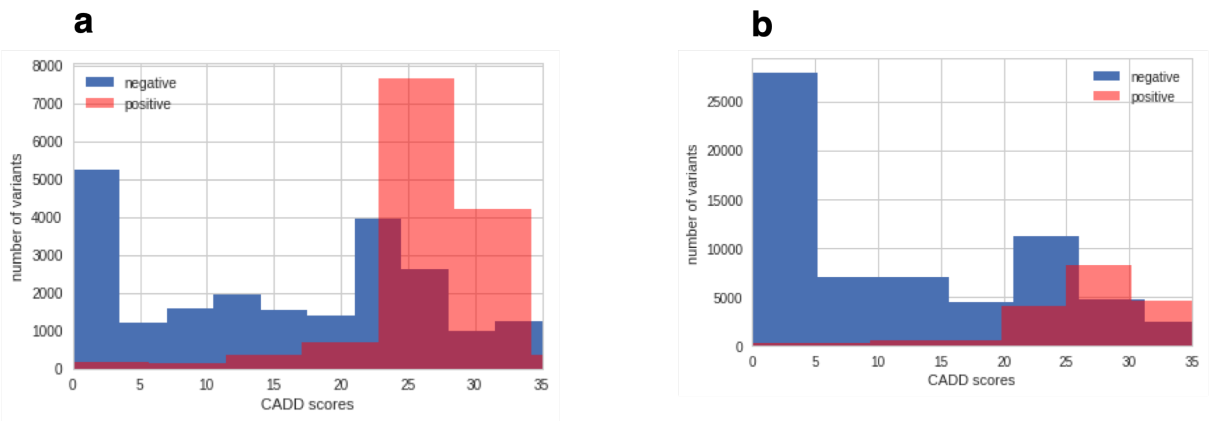

Supplementary Figure 18. Distribution of CADD scores on training data set. (a) Constrained genes with ExAC pLI ≥ 0.5; **(b)** Non-constrained genes with ExAC pLI < 0.5. Human-derived changes from the CADD database are used in training but CADD score itself is not a training feature.

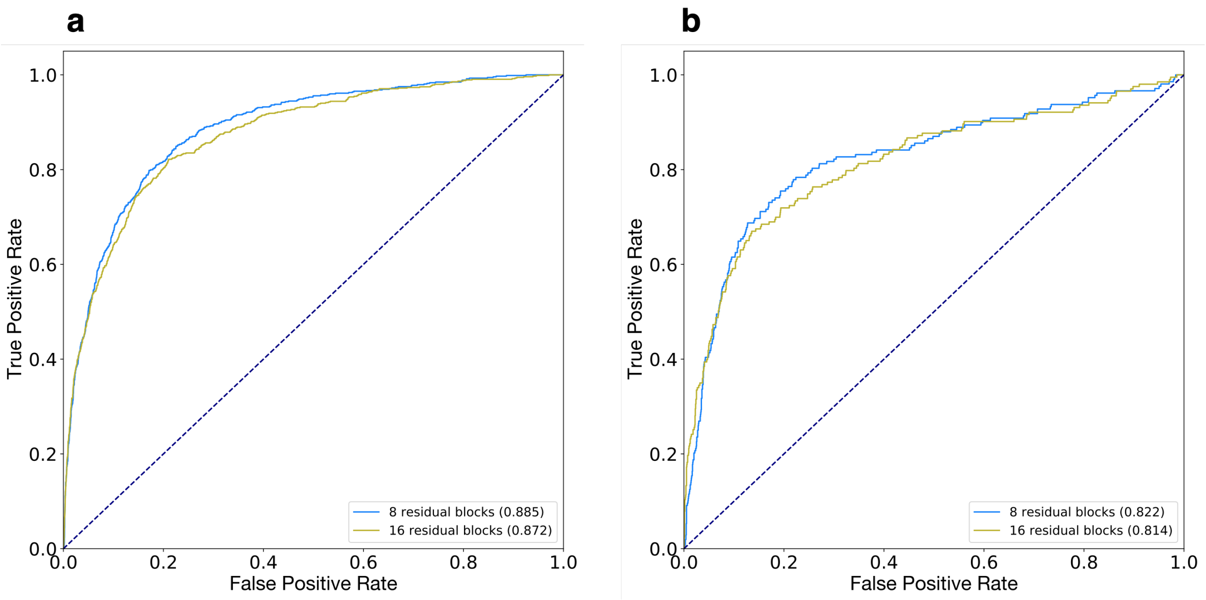

Supplementary Figure 19. ROC curves for the models with 8 and 16 residual blocks on the cancer somatic mutation data sets. The model with 8 residual blocks works better for both **(a)** constrained genes and **(b)** non-constrained genes regarding the AUROC.

Supplementary Tables

Supplementary Table 1. Performance comparison of different methods in VariBench dataset and Cancer hotspot dataset. The table indicated the AUC performance of different predictors in VariBench data and cancer hotspot data, genes are grouped as constrained gene (ExAC pLI ≥ 0.5) and non-constrained gene (ExAC pLI < 0.5).

	constrained genes			non-constrained genes		
Methods	VariBench dataset	Cancer hotspot dataset	difference	VariBench dataset	Cancer hotspot dataset	difference
MVP	0.959	0.912	(0.047)	0.917	0.850	(0.067)
MetaSVM	0.900	0.837	(0.063)	0.902	0.769	(0.134)
MetaLR	0.899	0.829	(0.069)	0.909	0.770	(0.139)
M-CAP	0.902	0.840	(0.062)	0.899	0.822	(0.077)
DANN	0.646	0.685	0.039	0.669	0.686	0.017
CADD	0.744	0.783	0.040	0.716	0.737	0.021
Eigen-phred	0.777	0.813	0.036	0.753	0.735	(0.018)
Polyphen2 HVAR	0.783	0.794	0.011	0.747	0.724	(0.023)
Mutation-Taster	0.724	0.759	0.034	0.722	0.751	0.028
FATHMM	0.876	0.766	(0.109)	0.895	0.738	(0.157)
fathmm-MKL	0.658	0.754	0.097	0.716	0.691	(0.025)
REVEL	0.926	0.886	(0.040)	0.914	0.823	(0.091)
MPC	0.812	0.837	0.025	0.671	0.819	0.147
VEST3	0.879	0.845	(0.034)	0.869	0.791	(0.078)
PrimateAI	0.718	0.796	0.078	0.697	0.806	0.109
UNEECON	0.868	0.896	0.028	0.793	0.821	0.027

Supplementary Table 2

Comparison of cases and controls in rate of synonymous *de novo* variants

	Number of synonymous variants	Rate per cases compared to controls
Autism spectrum disorder (ASD)	1026	1.027
Congenital heart disease (CHD)	701	1.049
Simons Simplex Collection unaffected siblings (controls)	483	N/A

Supplementary Note 1

Performance inflation in different datasets

Databases of pathogenic variants curated from the literature are known to have a substantial frequency of false positives. There are likely similar factors causing false positives across different databases. Therefore, dividing the datasets into training and testing data does not create truly independent data for performance assessment, and as a result, the AURC calculated from VariBench data is likely inflated for methods trained on these datasets, including MVP and other methods with best AUROC values. This is supported by results in Supplementary Figure 5: using cancer somatic mutation hotspots as positives, and randomly selected rare variants from DiscovEHR as negatives, the area under receiver operating characteristic curve (AUROC) of all methods trained by HGMD or UniProt is substantially decreased. Notably, MPC, which was trained on a small set of high-confidence ClinVar data, saw increased performance in cancer data, especially in non-constrained genes.

The results from *de novo* mutations provide further support. In Supplementary Figure 8, we estimated the precision of the optimal MVP score based on ROC curves with cancer and VariBench data, and used baseline precision (i.e. precision of "all missense") to bridge ROC and Precision-Recall calculation (see details below). The figure shows that the Precision-Recall point of optimal MVP score in *de novo* mutations is much closer to the estimated point based on cancer ROC curves than VariBench ROC curve in both constrained and non-constrained genes (Supplementary Figure 8).

The procedure to estimate precision for a method at a certain threshold based on ROC curves

Denote the number of all true positives (pathogenic variants in cases) in a *de novo* mutation data set as $\boldsymbol{P}$, the estimated number of true positive detected by all methods at any threshold (including estimation from "all missense" without prediction methods) as a set $\boldsymbol{\mathcal{P}}$, the number of all negatives (non-pathogenic variants in cases) in the de novo mutation data as $\boldsymbol{N}$, the number of true positives by a

method at a threshold as *TP*, the number of false positives by a method at a threshold as *FP*, and the baseline precision as ***B***, defined as:

$$B \equiv \frac{P}{P+N}$$

P + ***N*** is just the total number of *de novo* mutations in cases. We can estimate *B* by:

$$\hat{B} = \frac{\max(\boldsymbol{\mathcal{P}})}{P+N}$$

Therefore, **N / P** can be estimated as:

$$\frac{N}{P} = \frac{1}{1/\hat{B} \; - 1}$$

From the ROC curve, denote true positive rate (which is also called *recall* or *sensitivity*) as *TPR*, and false positive rate as *FPR*. We obtain *FPR* and *TPR* for a method at a certain threshold from cancer or VariBench ROC curves, and then use them to estimate number of true and false positives:

$$\widehat{TP} = P \cdot TPR$$

$$\widehat{FP} = N \cdot FPR$$

Therefore, the estimated precision of a method at a threshold based on ROC curve is:

$$\widehat{Precision} = \frac{\widehat{TP}}{\widehat{TP} + \widehat{FP}} = \frac{1}{1 + \frac{\widehat{FP}}{\widehat{TP}}} = \frac{1}{1 + \frac{FPR}{TPR} \cdot \frac{N}{P}} = \frac{1}{1 + \frac{FPR}{TPR} * (\frac{1}{\hat{B}} - 1)}$$
