# Supplementary material for: MVP predicts the pathogenicity of missense variants by deep learning
Source: Nat Commun. 2021 Jan 21;12:510. doi: 10.1038/s41467-020-20847-0 (PMC7820281; doi:10.1038/s41467-020-20847-0)
Supplement: Supplementary file 3 — Description of additional supplementary files [file 41467_2020_20847_MOESM3_ESM.pdf]

Description of Additional Supplementary Files

File Name: Supplementary Data 1.

Description: Estimated number of pathogenic missense de novo mutations using published methods by recommended thresholds. The table indicates their thresholds, estimated number of risk variants and positive predictive values in Congenital heart disease and Autism spectrum disorder data.

File Name: Supplementary Data 2.

Description: Features in the MVP model. The table lists details of features used in constrained genes model and non-constrained genes model grouped by different categories.

File Name: Supplementary Data 3.

Description: Summary statistics of training and testing data sets. The table indicates number of genes and variants from different data sets used in training and testing. Genes are grouped as constrained genes (ExAC pLI ≥ 0.5) and non- constrained genes (ExAC pLI < 0.5).

File Name: Supplementary Data 4.

Description: Number and percentage of genes and variants in testing datasets that are overlapped with genes used in training.

File Name: Supplementary Data 5.

Description: CHD de novo variants D-mis enrichment using different methods by various rank percentile thresholds. The table indicates rank percentile threshold for each method, number of variants in cases and controls passing the criteria, enrichment, two-sided binomial test p-value, estimated number of risk variants and positive predictive values and estimated recall.

File Name: Supplementary Data 6.

Description: ASD de novo variants D-mis enrichment using different methods by various rank percentile thresholds. The table indicates rank percentile threshold for each method, number of variants in cases and controls passing the criteria, enrichment, two-sided binomial test p-value, estimated number of risk variants and positive predictive values and estimated recall.

File Name: Supplementary Data 7.

Description: Percentage of CHD isolated cases by damaging variants. We define damaging missense variants using various rank percentile thresholds, for MetaSVM prediction we used recommended score of 0. The table indicates rank percentile threshold for each method, number of variants in cases and controls passing the criteria, enrichment, two-sided binomial test p-value, estimated

number of risk variants, positive predictive values, estimated recall, percentage of the cases explained by de novo loss of function variants and damaging missense variants with 95% confident interval.

File Name: Supplementary Data 8.

Description: Predicted pathogenic variants in isolated CHD cases. There are 235 predicted pathogenic variants, including 137 pathogenic missense variants by MVP score ≥ 0.75 and 98 loss-of-function variants which are likely to disrupt genes (e.g. frameshift, splicing site, nonsense, start/stop loss mutations). The genomic position (hg19) of each variant and the alternative alleles were indicated. The function predicted values of each variant was given by CADD, metaSVM, M-CAP, MPC and REVEL. The rank percentile of function predicted values of each variant was given by CADD_rank, metaSVM_rank, M-CAP_rank, MPC_rank, REVEL_rank and MVP_rank. The higher rank value, the more likely to be pathogenic. The ExAC pLI value indicates gene intolerance. We only considered constrained genes with value of pLI ≥ 0.5 and minor allele frequency (MAF) smaller than 1e-6 and non-constrained genes with value of pLI < 0.5 and MAF smaller than 1e-4.

File Name: Supplementary Data 9.

Description: CHD de novo missense variants with annotation. The genomic position (hg19) of each variant and the alternative alleles were indicated. The function predicted values of each variant was given by CADD, metaSVM, M-CAP, MPC and REVEL. The rank percentile of function predicted values of each variant was given by CADD_rank, metaSVM_rank, M-CAP_rank, MPC_rank, REVEL_rank and MVP_rank. The higher rank value, the more likely to be pathogenic. The ExAC pLI value indicates gene intolerance. We only considered constrained genes with value of pLI ≥ 0.5 and minor allele frequency (MAF) less than 1e-6 and non-constrained genes with value of pLI < 0.5 and MAF less than 1e-4.

File Name: Supplementary Data 10.

Description: ASD de novo missense variants with annotation. The genomic position (hg19) of each variant and the alternative alleles were indicated. The function predicted values of each variant was given by CADD, metaSVM, M-CAP, MPC and REVEL. The rank percentile of function predicted values of each variant was given by CADD_rank, metaSVM_rank, M-CAP_rank, MPC_rank, REVEL_rank and MVP_rank. The higher rank value, the more likely to be pathogenic. The ExAC pLI value indicates gene intolerance. We only considered constrained genes with value of pLI ≥ 0.5 and minor allele frequency (MAF) less than 1e-6 and non-constrained genes with value of pLI < 0.5 and MAF less than 1e-4.

File Name: Supplementary Data 11.

Description: SSC control de novo missense variants with annotations. The genomic position (hg19) of each variant and the alternative alleles were indicated. The function predicted values of each variant was given by CADD, metaSVM, M-CAP, MPC and REVEL. The rank percentile of function predicted values of each variant was given by CADD_rank, metaSVM_rank, M-CAP_rank, MPC_rank, REVEL_rank and MVP_rank. The higher rank value, the more likely to be pathogenic. The ExAC pLI value indicates gene intolerance, we only consider constrained genes with value of pLI ≥ 0.5 and

minor allele frequency (MAF) less than 1e-6 and non-constrained genes with value of pLI < 0.5 and MAF less than 1e-4.

File Name: Supplementary Data 12.

Description: Enriched pathways with FDR < 0.01 and odds ratio > 4 in either isolated cases or syndromic cases. Results are given by Enrichr using Reactome database. 136 genes in isolated CHD cases and 344 genes in syndromic cases with de novo missense variants of MVP rank score ≥ 0.75 are used respectively. P-value is computed using Fisher's exact test (one-tailed), while adjusted p-value is corrected by BH procedure for multiple tests.
