# Supplementary material for: MVP predicts the pathogenicity of missense variants by deep learning
Source: Nat Commun. 2021 Jan 21;12:510. doi: 10.1038/s41467-020-20847-0 (PMC7820281; doi:10.1038/s41467-020-20847-0)
Supplement: Supplementary file 16 — Reporting Summary [file 41467_2020_20847_MOESM16_ESM.pdf]

nature research

Corresponding author(s): Yufeng Shen

Last updated by author(s): Dec 7, 2020

Reporting Summary

Nature Research wishes to improve the reproducibility of the work that we publish. This form provides structure for consistency and transparency in reporting. For further information on Nature Research policies, see our Editorial Policies and the Editorial Policy Checklist.

Statistics

For all statistical analyses, confirm that the following items are present in the figure legend, table legend, main text, or Methods section.

n/a	Confirmed	
☐	☒	The exact sample size (n) for each experimental group/condition, given as a discrete number and unit of measurement
☒	☐	A statement on whether measurements were taken from distinct samples or whether the same sample was measured repeatedly
☐	☒	The statistical test(s) used AND whether they are one- or two-sided<br>*Only common tests should be described solely by name; describe more complex techniques in the Methods section.*
☒	☐	A description of all covariates tested
☐	☒	A description of any assumptions or corrections, such as tests of normality and adjustment for multiple comparisons
☐	☒	A full description of the statistical parameters including central tendency (e.g. means) or other basic estimates (e.g. regression coefficient) AND variation (e.g. standard deviation) or associated estimates of uncertainty (e.g. confidence intervals)
☐	☒	For null hypothesis testing, the test statistic (e.g. F, t, r) with confidence intervals, effect sizes, degrees of freedom and P value noted<br>*Give P values as exact values whenever suitable.*
☒	☐	For Bayesian analysis, information on the choice of priors and Markov chain Monte Carlo settings
☒	☐	For hierarchical and complex designs, identification of the appropriate level for tests and full reporting of outcomes
☐	☒	Estimates of effect sizes (e.g. Cohen's d, Pearson's r), indicating how they were calculated

Our web collection on statistics for biologists contains articles on many of the points above.

Software and code

Policy information about availability of computer code

Data collection	we used data from recent publications and public or commercial databases, all properly cited in the manuscript. We include a section "Data availability" in the manuscript describing the sources of the data sets used in the study. We did not generate new original data.
Data analysis	We used python (version 3.6) and R (version 3.2) for data analysis. Scripts are available on github: https://github.com/ShenLab/missense . It is also described in the manuscript.

For manuscripts utilizing custom algorithms or software that are central to the research but not yet described in published literature, software must be made available to editors and reviewers. We strongly encourage code deposition in a community repository (e.g. GitHub). See the Nature Research guidelines for submitting code & software for further information.

Data

Policy information about availability of data

All manuscripts must include a data availability statement. This statement should provide the following information, where applicable:

- Accession codes, unique identifiers, or web links for publicly available datasets
- A list of figures that have associated raw data
- A description of any restrictions on data availability

1. Precomputed MVP pathogenicity score for all possible missense variants in canonical transcripts on human hg19 can be downloaded without restriction from: https://figshare.com/articles/dataset/Predicting_pathogenicity_of_missense_variants_by_deep_learning/13204118.
2. The training and testing data can be accessed through
a. DiscovEHR: http://www.discovehrshare.com/downloads
b. VariBench: http://structure.bmc.lu.se/VariBench/substitutionsall.php

c. HGMD: http://www.hgmd.cf.ac.uk/ac/index.php
d. UniProt: https://www.uniprot.org/docs/humpvar
e. ClinVar: https://ftp.ncbi.nlm.nih.gov/pub/clinvar/vcf_GRCh37/

Field-specific reporting

Please select the one below that is the best fit for your research. If you are not sure, read the appropriate sections before making your selection.

- [x] Life sciences
- [ ] Behavioural & social sciences
- [ ] Ecological, evolutionary & environmental sciences

For a reference copy of the document with all sections, see nature.com/documents/nr-reporting-summary-flat.pdf

Life sciences study design

All studies must disclose on these points even when the disclosure is negative.

Sample size	N/A. This study does NOT deal with human subjects for estimating the effect size of a treatment. The statistical analyses have been reviewed based on the evidence from data that are available for each comparison.
Data exclusions	N/A. We did not exclude data as described in the study.
Replication	N/A. This is not an association study. The performance of the new method was evaluated by independent testing data sets from different sources.
Randomization	N/A. This is not a trial study.
Blinding	N/A.

Reporting for specific materials, systems and methods

We require information from authors about some types of materials, experimental systems and methods used in many studies. Here, indicate whether each material, system or method listed is relevant to your study. If you are not sure if a list item applies to your research, read the appropriate section before selecting a response.

Materials & experimental systems

n/a	Involved in the study	
x		Antibodies
x		Eukaryotic cell lines
x		Palaeontology and archaeology
x		Animals and other organisms
x		Human research participants
x		Clinical data
x		Dual use research of concern

Methods

n/a	Involved in the study	
x		ChIP-seq
x		Flow cytometry
x		MRI-based neuroimaging
